# Hypoglycemic Effects of Plant Flavonoids: A Review

**DOI:** 10.1155/2021/2057333

**Published:** 2021-12-08

**Authors:** Foo Sok Yen, Chan Shu Qin, Sharryl Tan Shi Xuan, Puah Jia Ying, Hong Yi Le, Thiviya Darmarajan, Baskaran Gunasekaran, Shamala Salvamani

**Affiliations:** ^1^Faculty of Applied Sciences, UCSI University, Jalan Menara Gading 1, Taman Connaught Cheras, Kuala Lumpur 56000, Malaysia; ^2^Division of Applied Biomedical Science and Biotechnology, School of Health Sciences, International Medical University, Bukit Jalil, Kuala Lumpur 57000, Malaysia

## Abstract

Diabetes mellitus is a metabolic disorder with chronic high blood glucose levels, and it is associated with defects in insulin secretion, insulin resistance, or both. It is also a major public issue, affecting the world's population. This disease contributes to long-term health complications such as dysfunction and failure of multiple organs, including nerves, heart, blood vessels, kidneys, and eyes. Flavonoids are phenolic compounds found in nature and usually present as secondary metabolites in plants, vegetables, and fungi. Flavonoids possess many health benefits such as anti-inflammatory and antioxidant activities, and naturally occurring flavonoids contribute to antidiabetic effects.Many studies conducted *in vivo* and *in vitro* have proven the hypoglycemic effect of plant flavonoids. A large number of studies showed that flavonoids hold positive results in controlling the blood glucose level in streptozotocin (STZ)-induced diabetic rats and further prevent the complications of diabetes. The future development of flavonoid-based drugs is believed to provide significant effects on diabetes mellitus and diabetes complication diseases. This review aims at summarizing the various types of flavonoids that function as hyperglycemia regulators such as inhibitors of *α*-glucosidase and glucose cotransporters in the body. This review article discusses the hypoglycemic effects of selected plant flavonoids namely quercetin, kaempferol, rutin, naringenin, fisetin, and morin. Four search engines, PubMed, Google Scholar, Scopus, and SciFinder, are used to collect the data.

## 1. Introduction

Diabetes mellitus (DM) is a significant concern affecting the world, with a tremendous increase in cases over the years. The prevalence of DM has been increasing worldwide, especially within countries of low- to middle-income status. According to the global report from World Health Organization (WHO), the number of people living with DM has nearly quadrupled in the last 30 years, rising from 108 million in 1980 to 422 million in 2014. In the year 2012, 2.2 million deaths were due to hyperglycemia, and over 1.5 million deaths were closely attributed to DM. By 2030, DM is predicted to be the world's seventh major cause of death [1]. DM has also been causing severe organ failure over the years rapidly becoming one of the noncommunicable diseases causing a rapid increase in mortality rates [2]. Complex metabolic disorder in the endocrine system is the cause of DM, and it was characterized by hyperglycemia and hindrance in the metabolism of protein, carbohydrates, and lipid resulting from either impairment or destruction of insulin action and insulin-secreting pancreatic *β*-cells in target tissues, or both insulin-dependent diabetes mellitus (IDDM, type 1) and noninsulin-dependent diabetes mellitus (NIDDM, type 2), which are the two main types of diabetes. Type 11 diabetes is an autoimmune disorder in which the insulin-secreting cells were attacked and destroyed by local inflammatory mediators [3, 4]. The most encountered form of diabetes is NIDDM as it accounts for more than 80% of the total case of DM [5]. It leads to pancreatic *β*-cell failure, insulin resistance, and progressive hyperglycemia.

Besides, chronic hyperglycemia resulting from DM has become a serious problem due to its association with severe long-term health complications such as dysfunction and failure of multiple organs, including nerves, heart, blood vessels, kidneys, and eyes [6]. One of the many therapeutic methods that helps reduce postprandial hyperglycemia is to inhibit the carbohydrate-hydrolysing enzyme, glucosidase, found in the intestine that plays a huge role in facilitating the uptake of glucose by breaking down carbohydrates before monosaccharide absorption. Alpha-glucosidase inhibitors also play an important role in reducing the insulin peaks and postprandial glycemia by delaying the absorption of ingested carbohydrates [7]. It is found that the long-term use of antidiabetic drugs can cause various adverse side effects, which can be a crucial problem to eradicate [8]. For example, the administration of alpha-glucosidases and alpha-amylases can result in gastrointestinal-related side effects, such as diarrhea, flatulence, and abdominal discomfort [9]. Besides that, acarbose and thiazolidinediones, which are the drugs used to treat type 2 diabetes, which was reported in 2006, have also produced side effects resulting in hepatic failure and injury [10]. Scientists believe that an effective control of blood glucose levels can prevent and reduce the clinical complication of DM, thus shifting the therapeutic development process from antidiabetic drug discovery to alternative treatment approaches, which have minimal side effects [11].

In recent years, many natural compounds originating from plants have shown antidiabetic activity, along with promoting human health. The bioactive compounds found in the medicinal plants can be studied as an alternative therapeutic approach as they are also relatively cheap and affordable, which are the factors to be considered for drug development approaches. These compounds are safe to be consumed in daily diets and have shown positive outcomes in treating many different diseases as well as reducing the risks of future diseases [5]. The bioactive compounds that are isolated from plants have shown positive effects in both *in vivo* and *in vitro* studies, resulting in hypocholesterolemia, hypotensive, hypoglycemic, and antioxidant effects [12, 13]. Therefore, this review article aims at summarizing several significant studies that have revealed the antidiabetic potential of selected plant flavonoids and their molecular mechanism in treating and preventing DM.

## 2. Flavonoids

The term flavonoids was derived from the Latin word *flavus*, which means yellow [14]. Flavonoid compounds are ubiquitous in plants and have plenty of polyphenolic compounds in the human diet [13]. The phytochemical compound cannot be synthesized by animals and humans. The phenolic compounds extracted from plant flavonoids have proven its therapeutic potential and thus attracted the interest of scientists [15]. Furthermore, there are more than 5000 flavonoid compounds that have shown numerous positive effects, which have been identified and isolated from different plants [3]. According to research and clinical studies, flavonoids have shown their beneficial effects in the prevention, alleviation, and treatment of numerous degenerative and viral diseases, such as cancers, obesity, cardiovascular diseases, diabetes, and other age-related diseases [16, 17]. In addition, it also acts as an antioxidant to modulate oxidative stresses in the body by neutralizing the effects of reactive oxygen and nitrogen, thereby preventing various diseases.

Flavonoids consist of a large group of polyphenolic compounds with a variable phenolic structure and present as a benzo-*γ*-pyrone structure [18]. Skeleton benzo-*γ*-pyran (C6-C3-C6) is the common chemical structure for all flavonoids. Flavonoids are made up of a fifteen-carbon skeleton and assembled in two benzene rings, which are denoted as A and B, with a connected heterocyclic pyrene ring denoted as C. Activities of flavonoids are structural-dependent, including the degree of hydroxylation, type of conjugation, degree of polymerization, pattern of substitution, and structural class [19].

Flavonoids such as flavones, flavanols, isoflavonoid, anthocyanins, chalcone, and flavanones are classified into different classes due to the differences in their structure. Each class of flavonoids also possesses different patterns of substitution of the C ring and also its levels of oxidation. However, the differences only at the A and B rings are categorized under the same class, but different flavonoid subclasses [20]. The types of flavonoids also differ from different natural sources as different classes of flavonoids are derived from different plants.

### 2.1. Absorption and Bioavailability of Flavonoids

Most flavonoids except for the catechin subclass are present as *β*-glycosides in plants bound to sugars. The absorption of the food-derived dietary flavonoids into the gut depends on their physicochemical properties such as solubility, acid dissociation constant (pKa), the size of molecule, configuration, and lipophilicity. Zagrean-Tuza et al. reported that the absorption of quercetin glycosides is determined by their sugar moiety [21]. The low molecular weight and high lipophilicity of flavonoid aglycones enable them to easily penetrate the lumen into bloodstream via the monolayer. However, flavonoid glycosides with a higher molecular weight and hydrophilicity are likely to limit their absorption.

During absorption, flavonoids either are absorbed in the small intestine or progress to the colon. It largely depends on the flavonoid structure, whether it is a glycoside or an aglycone. Epithelial cells are only able to absorb the liberated aglycone through passive diffusion because of their hydrophobic characteristic [22]. If flavonoid glycoside is not absorbed in the small intestine, it can be metabolized in the large intestine by the colonic microflora, which is the first stage in the flavonoid-glycoside metabolism into its aglycone. The human colonic microflora is composed primarily of more than 400 species of bacteria. Furthermore, Schloissnig et al. found that *β*-glucosidase, which is an enzyme in charge of the hydroxylation of glycosidic bonds, is synthesized by the Bacteroides genus such as *B. uniformis*, *B. ovatus*, and one strain of *B. distasonis* [23]. A study conducted by Lu et al. observed that the rate of glucuronidation by *β*-glucuronidase relied on the conjugation position [24]. Since the absorption capacity of the colon is lower than the small intestine, the absorption of the glycosides is relatively low. Furthermore, after being absorbed in the small intestine, the flavonoids are further conjugated by the largest metabolizing organ, the liver, through processes such as methylation, sulfation, glucuronidation, and sulfation into smaller phenolic and hydrophilic compounds for easier excretion and distribution in the bloodstream [25].

### 2.2. Flavonoids as Hyperglycemia Regulators

Dietary flavonoids are well known for their benefits concerning glucose homeostasis. This is because studies have shown that flavonoids are capable of inhibiting carbohydrate digestions and glucose absorptions, along with the regulation of insulin secretions via multiple signaling pathways [26]. This is achieved as flavonoids can simply inhibit carbohydrate-digesting enzymes and glucose transporters, which aids in achieving normoglycemia within the blood circulation. These mechanisms are relatively important as a therapeutic approach towards DM.

#### 2.2.1. Inhibitors of *α*-Glucosidase

**Figure 1:**
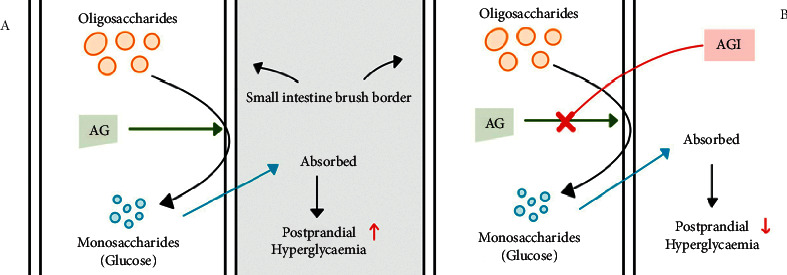
Mechanism of action of *α*-glucosidase inhibitors. A: absence of *α*-glucosidase inhibitor; B: presence of *α*-glucosidase inhibitor. AG: *α*-glucosidase; AGI: *α*-glucosidase inhibitor (adapted from Arungarinathan et al. [29]).

*α*-Glucosidase is among the most essential membrane-bound enzyme in carbohydrate digestion found in the small intestine epithelium. The activities of *α*-glucosidase correlate to maltase-glucoamylase (MGAM) and sucrase-isomaltose (SI), which are located in the intestinal brush border [27]. Glucose is released from the non-reducing end through the hydrolysis of linear *α* − 1 ⟶ 4 and branched *α* − 1 ⟶ 6 linkages of oligolinkages [28]. The inhibition of *α*-glucosidase can delay the degradation of complex sugars into glucose, which helps delay the absorption of glucose in the small intestine, eventually decreasing postprandial blood sugar levels (Figure 1).

Hong et al. showed that the flavonoids, mainly astragalin, rutin, isoquercetin, and kaempferol-3-O-rutinoside in *Morus atropurpurea* leaves, can suppress *α*-glucosidase activity [30]. Among these four flavonoids, rutin and astragalin have shown remarkable *α*-glucosidase inhibitory activities of IC_50_ values (13.19 ± 1.10 and 15.82 ± 1.11 *μ*M, respectively). In addition, Zeng et al. have reported that *α*-glucosidase activity was significantly reduced via competitive inhibition by apigenin [31]. Furthermore, molecular simulation has shown that apigenin is bound to a site near the *α*-glucosidase active site, which could trigger the channel closure to restrict the access of substrate, ultimately leading to *α*-glucosidase inhibition. Geranylated flavonoids, namely, 3′-O-methyl-5′-O-methyldiplacone, 4′-O-methyldiplacone, 3′-O-methyldiplacone, 4′-methoxyflavanone, mimulone, 4′-O-methyldiplacol, and 3′-O-methyldiplacol, were isolated from *Paulownia tomentosa* and revealed a significant inhibitory effect on *α*-glucosidase with IC_50_ values ranging from 2.2 to 78.9 *μ*Μ [32]. Besides that, rutin, kaempferol hexose, and catechin were the most abundant flavonoids found in the extraction of *R. roxburghii* fruits dispersion. Among these components, catechin showed the greatest inhibitory effects on *α*-glucosidase with the highest IC_50_ value [33].

#### 2.2.2. Inhibitors of Glucose Cotransporter

Glucose derived from carbohydrate-rich diet is a major essential metabolic substrate that gets absorbed from the brush border of the gut to targeted cells via the bloodstream. In the process, integral transport proteins function as shuttles in the processes of transferring glucose through plasma membranes. These glucose cotransporters can be classified into two categories: (i) sodium-dependent glucose transporters (SGLT) and (ii) facilitative glucose transporters (GLUT) [34].

The SGLT is an energy-dependent sodium/glucose cotransporter that plays an essential role in the absorption of glucose. SGLTs are in the gut and in the proximal tubules of the kidney allowing the absorption of glucose. These carrier proteins carry glucose through active transport [35]. SGLT uses sodium ions movement down its electrochemical gradient to transport the glucose to the targeted cells. SGLT1 and SGLT2 are the isoforms of SGLT, with SGLT2 having a lower affinity, and are located almost exclusively in the kidney where the transportation of plasma glucose from the glomerular filtrate takes place [36]. SGLT2 has become a major drug target because the inhibition of SGLT2 can reduce the glucose reabsorption by increasing glucose excretion in the urine and simultaneously controlling the blood glucose level. Formononetin compounds derived from *Sophora flavescens*, which are also often used in traditional Chinese medicines, showed potent interruption activity on SGLT2 [37]. On the other hand, although SGLT1 is of high affinity and located primarily in the small intestine, it is also expressed in the kidneys to recover any remaining glucose with the aim of preventing glucose loss in the urine. Schulze et al. reported that quercetin 4′-O-glucoside (Q4′glc), flavonoid from onion, showed the strongest inhibition effect on SGLT1 in hyperglycemic mice [38].

SLC2 genes are responsible for encoding the GLUT proteins, which are responsible for transporting monosaccharides, small carbon compounds, and polyols through plasma membranes using the diffusion gradient. GLUTs exhibit different substrate specificities, tissue expression profiles, and kinetic properties. Many members of the GLUT family express themselves differently. GLUT1 is expressed almost ubiquitously in all normal tissues often in association with one or more additional GLUT isoforms to maintain the basal glucose supply. GLUT2 and GLUT3 are involved in essential processes such as pancreatic insulin secretion [39] and neuronal glucose [40]. Several flavonoids have shown their GLUT2 inhibition effects with one of the recent studies revealing that three homoisoflavonoids of the sappanin type (SAP) purified from the *Polygonatum odoratum* (Mill*.) Druce* roots, another well-known Chinese traditional medicine, showed potent GLUT2 inhibitory effects [20]. However, GLUT4, mostly found in the heart and brain, is accountable for insulin-stimulated glucose transport. Not forgetting, GLUT7, which is of high affinity for both glucose and fructose, expressed high levels in the ileum responsible for sugar uptake at the end of a meal when sugar concentrations gradually decrease [41].

## 3. Flavonoids of Various Plants and Their Hypoglycemic Effects

Over the years, flavonoids from plant sources have been exhibiting antidiabetic activity through several mechanisms both *in vitro* and *in vivo*. The common flavonoids with hypoglycemic properties include quercetin, kaempferol, rutin, naringenin, fisetin, and morin. Plants such as *Fagopyrum tataricum*, *Gynura procumbens,* and *Tetracera indica* that consist of flavonoids have been reported to possess significant hypoglycemic effects as well [42].  Table 1 summarizes the mode of action of the reported plant flavonoids.

### 3.1. Quercetin

Quercetin (3,5,6,3′,4′-pentahydroxyflavone) is a naturally occurring dietary flavonoid that is commonly found in plants and fruits. Some of the many plants, which consist of quercetin, are green leafy vegetables, seeds, nuts, broccoli, onions, olive oil, pepper, tea leaves, and red wines, as well as fruits such as apples, cherries, and blueberries [50]. Numerous research studies have demonstrated that quercetin from plant sources was effective in reducing blood glucose levels. It can regulate glucose absorption that leads to glucose homeostasis through the interaction with various molecular targets in skeletal muscles, pancreas, small intestine, and liver in the body [43]. The hypoglycemic action of quercetin involves the inhibition of intestinal carbohydrate digestion, glucose transporter activity and glucose production in the liver, and improvement of glucose utilization in peripheral tissues as well as the protection against pancreatic islet damage [44].

Quercetin-rich extracts from *Vaccinium vitis-idaea* L.'s berries were proven to exert antidiabetic activity via the stimulation of insulin-independent AMP-activated protein kinase (AMPK) signaling pathway as well as subsequent basal uptake of glucose in skeletal muscle cells [45]. Eid et al. evaluated the quercetin's molecular mechanism of action in L6 myotubes and found that quercetin improved glucose uptake through the AMPK signaling pathway in muscle cells [46]. In skeletal muscles, the activation of the AMPK pathway can augment glucose intake by the translocation of glucose transporter 4 (GLUT4) to the plasma membrane. On the other hand, the AMPK pathway reduces hepatic glucose production through the downregulation of enzymes involved in gluconeogenesis such as glucose-6 phosphate and phosphoenolpyruvate carboxylase [47]. Moreover, quercetin has the potential to suppress glucoside uptake activity that is mediated by SGLT 1 in the human intestinal epithelial cells (Caco-2) through the interaction with transporters. Quercetin was proven to lower blood glucose levels by inhibiting the *α*-glucoside activity that is involved in carbohydrate digestion [7]. It was found that both sucrase and maltase activities are inhibited significantly after quercetin treatment *in vitro* and *in vivo* at 50 mg/kg [47]. Furthermore, Rifaai et al. reported that quercetin could protect against islet beta-cell damage and support the regeneration of beta-cells [48].

The hypoglycemic effect of quercetin was studied in animal models. In addition, Ahmad et al. [79] reported that the quercetin remarkably lowered the plasma glucose levels in streptozotocin-induced diabetic rats on the 21st day of treatment when compared to the control rats. Another study also revealed that the consumption of a diet containing quercetin at different concentrations (0.04% and 0.08%) for six weeks significantly declined blood glucose levels up to 15% and 31%, respectively, in type 2 diabetic mice as compared to the control group without quercetin [80]. An *in vivo* study has claimed that quercetin downregulated the oxidative damage and increased glucose uptake in rats via AMPK activation [81]. Quercetin also has the ability to reduce intestinal glucose uptake and decrease postprandial blood glucose levels in diabetic mice through the inhibition of the GLUT2 glucose transporter [82]. Besides, *Allium cepa* L. peel extracts containing quercetin as their major flavonoids were proven to attenuate insulin resistance and hyperglycemia in STZ-induced diabetic rats that consumed a high-fat diet through the upregulation of glucose uptake in peripheral tissues as well as the downregulation of hepatic gene expression in inflammation [49]. Therefore, it is suggested that quercetin can be further developed as a natural antidiabetic agent. High contents of quercetin are commonly found in many plants including *Allium fistulosum*, *Calamus scipionum*, *Camellia sinensis*, *Capsicum annum,* and *Euonymus alatus* [50].

### 3.2. Kaempferol

Kaempferol (3,5,7-trihydroxy-2-[4-hydroxyphenyl]-4H-1-benzopyran-4-one), also known as kaempferol-3, kaempferide, and kaempferol flavanol, is a major flavonoid aglycone that can be isolated from plants [83]. It consists of the following characteristics: high antioxidant activity, anti-inflammatory activity, anticancer activity, and antidiabetic properties [84]. The ingested kaempferol will be absorbed by the small intestine, and then, it will be further broken down into glucurono- and sulfo-conjugated forms [85].

Kaempferol may stimulate the secretion of insulin and reduce glucose absorption in the small intestine or regulate blood glucose levels. Al-Numair et al. studied the effect of the concentration of kaempferol (50, 100, and 200 mg/kg) on plasma glucose levels in normal and STZ-induced diabetic rats [86]. The oral administration of kaempferol does not significantly alter the blood plasma glucose in normal rats; however, it significantly decreased the plasma glucose levels in diabetic rats after 45 days of treatment. Nevertheless, it also proved to increase the plasma insulin of diabetic rats. It showed the maximum hyperglycemic effect at the highest concentration (200 mg/kg) of treatment. In a study conducted by Peng et al., it is reported that kaempferol showed a potent inhibitory effect on *α*-glucosidase [51]. In the CD spectra analysis, the addition of kaempferol into *α*-glucosidase increased the tendency of *α*-helix and random coil contents (from 30.8% to 34.2% and from 27.6% to 29.8%). It suggested that kaempferol reacts with *α*-glucosidase and forms a complex, kaempferol-*α*-glucosidase. Besides, Zhang and Liu [52] have also evaluated the insertion of kaempferol to the human islets that are exposed to chronic hyperglycemia, and the activation of caspase-3 is significantly reduced. Kaempferol is capable of protecting beta-cells against hyperglycemia-induced beta-cell toxicity; it could also restore Bcl-2 protein expression in beta-cells and islets. Alkalidy et al. investigated the expression of AMPK and GLUT4 proteins in skeletal muscle and adipose tissue of HF diet-fed mice [53]. The expressions of AMPK and GLUT4 are significantly decreased in the skeletal muscle and adipose tissue of obese mice, whereas the treatment with 0.05% kaempferol increased the expression. Besides, kaempferol has been shown to have potent antidiabetic properties, with a high potential to be developed as antidiabetic drugs [87]. Fruits and vegetables such as tomatoes, grapes, broccoli, cabbage, and kale also contain the composition of kaempferol. Moreover, it is also found in medicinal plants, such as *Ginkgo biloba, Tilia spp, and Sophora japonica* [54]. However, the data related to the long-term effects and toxicity levels of kaempferol intake on the human body are insufficient. Thus, there is a need to carry out more clinical and in *vivo* studies varying the concentration of kaempferol [88].

### 3.3. Rutin

The natural rutin (3′,4′5,7-tetrahydroxy-flavone-3-rutinoside) is a citrus flavonoid glycoside that is abundantly found in plants. Rutin is also termed quercetin-3-rutinoside, rutoside, and sophorin. The name “rutin” comes from the plant *Ruta graveolens*, which contains rutin [89]. In recent years, rutin has appeared to be widely used as an additive in health supplements and medicine [55]. Rutin has many benefits, such as powerful antioxidants, radical scavenging effects, antidiabetic effects, and anti-inflammatory effects [90]. In common, low levels of antioxidants in blood show a higher risk factor for the development of chronic disease, and antioxidants are important in preventing DM [91]. After the rutin is ingested into the human body, it can be degraded into small metabolites by intestinal bacteria. Initially, rutin is metabolized into quercetin 3-O-glucose by losing rhamnose, and then, it will lose glucose molecules and convert into leucocyanidin [56].

Numerous studies have proven that rutin inhibits two enzymes that catalyze the digestion of carbohydrates, *α*-glucosidases, and *α*-amylase. The inhibition of these enzymes will block the small intestine from absorbing glucose molecules and hence prevents the sharp rise of blood glucose levels [92]. An *in vivo* study conducted by Ahmed et al. demonstrated a biochemical study of oral glucose tolerance test (OGTT) in normal and diabetic rats [93]. In the diabetic mice treated with rutin, the liver glycogen and serum insulin levels significantly fall off. Niture et al. reported that rutin decreased plasma glucose, glycosylated hemoglobin, serum tumor necrosis factor-alpha (TNF-alpha), interleukin-6 (IL-6), and high-density lipoprotein (HDL) in diabetic rats [57]. Rutin treatment also improved the structure of the islet cells. A study demonstrated that the increased glucose uptake in the muscle of rats in response to insulin is driven through signaling via the insulin receptor and phosphoinositide 3-kinase (PI3K). PI3K in the signaling pathways of rutin, activating several proteins such as protein kinase B (PKB), akt substrate of 160 kDa (AS160), and protein kinase C (PKC). These proteins significantly translocate the GLUT4 from an intracellular pool to the plasma membrane [94]. With no doubt, it is understood that the pancreatic beta-cells play a crucial role in insulin secretion, and insulin is released into the bloodstream when the blood glucose is high. Insulin promotes glycolysis, and the excess glucose is removed from blood [95]. However, insulin secretion and insulin resistance are dependent on calcium homeostasis. The changes in calcium concentrations may activate voltage-operated calcium channels, and the secretion of insulin highly depends on voltage-activated Ca^2+^ influx [58]. In a recent study, Kappel et al. have carried out *in vitro* studies on the uptake of calcium ions to study the mechanism of action of rutin on insulin secretion [59]. It is reported that rutin can alter Ca^2+^ uptake in isolated pancreatic islets, followed by an increase in the uptake of Ca^2+^. Tiwari et al. have documented those previous studies that showed rutin is safe at a concentration of 2000 mg/kg and the intake of rutin from dietary sources will not cause any toxicity effects [61]. Rutin is widely distributed in plants, for example, *Ruta graveolens*, *Morus alba*, asparagus, and buckwheat [62].

### 3.4. Naringenin

Naringenin (4,5,7-trihydroxy-flavanone) is a dietary flavonoid that is widely found in citrus and grapefruits. Several studies on naringenin demonstrated its antidiabetic, antidyslipidemia, antiatherogenic, and anti-inflammatory properties. The hypoglycemic effects of naringenin was examined both *in vivo* and *in vitro*. In an *in vitro* study, naringenin was found to ameliorate the effects of fructose and palmitate-induced insulin resistance by improving glucose uptake through insulin stimulation and translocation of glucose transporter GLUT4 in L6 myotubes and skeletal muscles via AMPK activation [67]. Zygmunt et al. studied the antidiabetic activity of naringenin and found that naringenin enhanced skeletal muscle glucose uptake via AMPK activation [63]. The *Sambucus nigra* L. (elderflower) constitutes naringenin as one of its major bioactive components significantly increases the uptake of glucose in primary porcine myotube cultures from animals [96]. Bhattacharya et al. have also reported on the effects of the naringenin on pancreatic *β*-cells and have shown that naringenin managed to enhance the secretion of insulin via glucose stimulation and protect the beta-cells from apoptosis [64].

Furthermore, the outcomes of naringenin treatment on streptozotocin-induced animal models were investigated in several *in vivo* studies. Ortiz-Andrade et al. reported on the hypoglycemic properties of the naringenin and have revealed that short-term treatment for five days with naringenin can significantly reduce the plasma glucose levels in streptozotocin-nicotinamide-induced diabetic rats [65]. A study that investigated the effects of naringenin extracted from orange peel has revealed that naringenin can enhance insulin receptor beta-subunit, GLUT4, and tissue insulin sensitivity in STZ/NA-induced diabetic rats [97]. Also, another study on STZ-induced diabetic rats that were fed with a high-fat diet and treated with naringenin showed that there is a significant reduction in postprandial blood glucose levels by the inhibition of *α*-glucosidase activity in the intestine that prolonged the carbohydrates' absorption in rats [66]. Moreover, a study performed on the overweight human subjects proved the reduction in plasma glucose levels after the consumption of citrus polyphenol extract containing naringenin for 12 weeks [68]. Nevertheless, naringenin was also found to exhibit hypoglycemic effects by the inhibition of gluconeogenesis via the upregulation of AMPK, which thereby gave a similar antidiabetic effect as metformin, a type of drug that is designed to treat DM [98]. This suggests that naringenin has the potential to be further explored as an alternative drug against DM. Naringenin can be extracted from various medicinal plants such as *Madagascar periwinkle*, *Catharanthus roseus,* and *Elaeodendron croceum* [99].

### 3.5. Fisetin

Fisetin (3,3′, 4′,7-tetrahydroxy flavone) is a structural-related flavan-3-ol and presents in various types of plants such as apple, strawberry, grape, persimmon, cucumber, and onion at concentrations of 2–160 *μ*g/g [100]. Fisetin plays a huge role in pharmacological properties, which include anticancer, anti-inflammatory, antiproliferative, and anti-hyperglycemic activities [12]. Furthermore, fisetin possesses antidiabetic effects and can lower down methylglyoxal-dependent protein glycation. It plays a part in limiting the complications of DM [101]. Glucose homeostasis can also be improved with fisetin by attenuating carbohydrate metabolism enzymes in the body. A result of a decrease in glycated hemoglobin (Hb1Ac) could be observed with the oral treatment of fisetin given in a dose of 10 mg/kg continuously for 30 days. Besides, the levels of blood glucose and the expression of the gluconeogenic genes protein level have been shown to decrease as well. At the same time, there is also an increase in the concentration of plasma insulin [70].

Many studies were conducted to investigate the antidiabetic properties of fisetin on rats. In an *in vivo* study, it was shown that the levels of NF-*κ*B p65, serum nitric oxide (NO), hemoglobin A1C (HbA1c), and blood glucose have been significantly reduced with the treatment of fisetin [69]. On the other hand, there is no indicative variation in the blood glucose levels shown on the control rats throughout the experimental period. Prasath et al investigated on the antidiabetic and antioxidant activity of fisetin on streptozotocin-induced rats [102]. The outcomes showed a significant reduction in blood glucose levels and an increase in the plasma insulin level upon the treatment with fisetin. It can be shown by the absence of urine sugar in fisetin-treated diabetic rats. In another report, fisetin has been proved to regulate hyperglycemia-mediated oxidative stress, the inflammation processes, and programmed cell death. These eventually showed an improvement in the development of diabetic cardiomyopathy in STZ-induced DM rats [72].

In an *in vitro* study, fisetin was reported to downregulate both glycogenolysis and gluconeogenesis. Fisetin can inhibit glucose, lactate, and pyruvate, which are released from endogenous glycogen. A concentration of Revise to 200 mM fisetin could bring the maximal inhibitions of glycogenolysis (49%) and glycolysis (59%). Meanwhile, 300 mM fisetin could inhibit gluconeogenesis from lactate and pyruvate or fructose [71]. Furthermore, high glucose biomolecule-induced cytokine production is inhibited in monocytes by fisetin, and this would eventually prevent DM [103]. Apart from that, fisetin plays an essential role in enhancing the activities of hexokinase. Reduction in the activities of glucose-6-phosphate dehydrogenase (G6PD) and glucose-6-phosphatase (G6Pase) has been shown with the aid of fisetin [73]. Fisetin is a flavonoid dietary ingredient that presents in the smoke tree (*Cotinus coggygria*) [71], and rich sources of fisetin can be found in plants such as *Butea frondosa*, *Gleditsia triacanthos*, *Quebracho Colorado*, *Curcuma longa*, *Rhus verniciflua*, *Acacia greggii*, and *Acacia berlandieri* [12].

### 3.6. Morin

Morin (3,5,7,2′,4′-pentahydroxyflavone) is a natural bioflavonoid and is also a major component of traditional medicinal herbs, which is primarily isolated from members of the *Moraceae* family. Morin appears as a light yellowish pigment and is a constituent of many herbs, fruits, and wine [74]. Besides, morin is also available in *Psidium guajava* (Indian guava). Guava contains antioxidant properties and is traditionally considered to be an effective antidiabetic plant [77]. Morin has many benefits to the human health, and it has been reported for its strong antioxidant and other pharmacological properties, which include antimutagenesis, anti-inflammation [104], cardioprotection, and anti-allergic [105]. Through *in vivo* and *in vitro* studies, many previous pieces of research have shown and proved the antioxidant, anti-inflammatory, and antiproliferative effects of morin [78, 106]. Furthermore, it has been shown that morin as an insulin-mimetic flavonoid has antidiabetic property [107].

Besides that, the levels of insulin in diabetic rats are dependently increased with fisetin as well. Potentiating the pancreatic secretion of insulin from the existing beta-cells or by its release from the bound form may be the possible mechanism for morin to take part in hypoglycemic action [75]. As the results for the diabetic rats treated with a higher dose of 30 mg/kg per day, there was a significant (*P* < 0.05) reduction in blood glucose levels, and at the same time, insulin levels were significantly (*P* < 0.05) increased compared to untreated diabetic rats [108]. In animal models, it is shown that the oral administration of morin for 30 days has significantly improved hyperglycemia, glucose intolerance, and insulin resistance. There was also a decrease in the levels of lipid peroxides and at the same time improvement in the antioxidant competence in diabetic rats treated with the morin [60].

Furthermore, morin is proven for its anti-inflammatory effects as it can effectively decrease the levels of inflammatory cytokines such as IL-6 and TNF-*α* [71]. Morin shows a systemic protective action and helps to reduce the negative side effects of several drugs, without interfering with their functions. In addition, *in vitro* and *in vivo* studies demonstrated that morin exhibits a very low toxicity level. Besides, its chronic administration is well tolerated. It is suggested that morin could be used, either alone or in combination with other drugs, to prevent many human pathologies [109]. Besides *Psidium guajava*, morin can also be found in seed weeds, almond (*Prunus dulcis*), fig (*Chlorophora tinctoria*) and Osage orange (*Maclura pomifera*) [60].

## 4. Limitation of Review

Despite the fact that plant flavonoids have been shown to have hypoglycemic effects on the human health, the recommended dietary intake of plant flavonoids from either real foods or supplements remains a concern. To increase the flavonoid content of the diet, flavonoids can be obtained by consuming vegetables and fruits. However, it is unknown how much vegetables and fruits should be consumed in order for them to exert a significant antidiabetic effect on the human body. On the other hand, dietary supplements are also another alternative sources of flavonoids. Flavonoids derived from plant sources contain not only flavonoids but also a sophisticated mixture of secondary plant metabolites. As a result, it is difficult to obtain a pure form of dietary supplement after extraction [73]. Furthermore, there is a scarcity of clear research on the effects of flavonoid supplements on the human health, specifically their metabolism in the body, recommended dosage, toxicity levels, and drug-nutrient interactions. Therefore, there is a need to collect more data from clinical and *in vivo* studies on different flavonoids before we affirm the application and suitable dosage of flavonoids in the antidiabetic supplement.

## 5. Conclusion

Many studies reported the common flavonoid compounds, which are found in plants to exhibit various therapeutic approaches in the prevention and development of DM, and other chronic diseases such as hypertension and obesity, which can lead to cardiovascular disease and hypercholesterolemia. Each flavonoid mentioned in this article shows a different mode of inhibition towards *α*-glucosidase and acts as an effective antidiabetic agent. The actual mode of action of flavonoids as an antidiabetic agent is prospectively related to their modulatory effect on regulating blood glucose levels by inhibiting glucose synthesis, promoting the uptake of glucose by muscle, enhancing secretion of insulin, reducing insulin resistance, promoting proliferation, and reducing apoptosis of the pancreatic beta-cell. The hypoglycemic effect of quercetin, kaempferol, rutin, and naringenin gave a better review as all of the flavonoids successfully lowered the blood glucose levels of patients who suffer from DM significantly. On the other hand, fisetin and morin also showed positive results for both *in vivo* and *in vitro* studies; however, these studies are being conducted in animal models, with a lack of data from the human trials. Since the incidence of DM worldwide is increasing rapidly, the demand for a safe and effective alternative treatment with antidiabetic activity is also greater.

## Figures and Tables

**Table 1:** The summary of selected plant flavonoids.

Flavonoids	Structure	Mode of actions	Plants
Quercetin	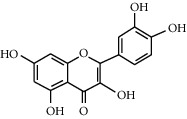	(1) Inhibits the activity of glucose transporter [43] (2) Enhances glucose uptake through the activation of the AMPK signaling pathway in skeletal muscle cells [44, 45] (3) Reduces hepatic glucose production [46] (4) An inhibitory effect on *α*-glucosidase [47] (5) Protects against pancreatic islet beta-cell damage and assists in the regeneration of beta-cells [48]	*Vaccinium vitis-idaea* L. [44] *Allium cepa* L. [49] *Allium fistulosum* [50] *Calamus scipionum* [50] *Camellia sinensis* [50] *Capsicum annum* [50] *Euonymus alatus* [50]

Kaempferol	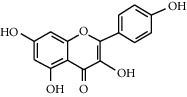	(1) Inhibitory effect on *α*-glucosidase [51] (2) Protects beta-cells against hyperglycemia-induced *β*-cell toxicity [52] (3) Improves the expressions of AMPK and GLUT4 [53]	*Ginkgo biloba* [54] *Tilia spp.* [54] *Sophora japonica* [54]

Rutin	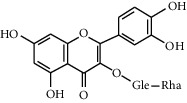	(1) Antioxidant properties [55] (2) Inhibitory effect on *α*-glucosidases and *α* amylase [56] (3) Enhances glucose uptake in the muscle cells [57] (4) Improves the uptake of Ca^2+^ ions and affects insulin secretion [58]	*Ruta graveolens* [59] *Morus alba* [59] *Amaranthus viridis* [60]

Naringenin	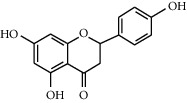	(1) Enhances glucose uptake through the activation of AMPK signaling pathway in skeletal muscle cells [61, 62] (2) Protects the *β*-cells from apoptosis [63] (3) Enhances insulin receptor beta-subunit, GLUT4, and tissue insulin sensitivity [64] (4) Inhibitory effect on *α*-glucosidases [65] (5) Inhibits gluconeogenesis through the upregulation of AMPK [66]	*Sambucus nigra* L. [67] *Madagascar periwinkle* [68] *Catharanthus roseus* [68] *Elaeodendron croceum* [68]

Fisetin	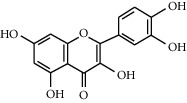	(1) Attenuates carbohydrate metabolism enzymes [69] (2) Downregulates glycogenolysis and gluconeogenesis [70] (3) Reduces activity of glucose 6 phosphate dehydrogenase (G6PD) and glucose 6-phosphatase (G6Pase) [71] (4) Inhibits high glucose biomolecule-induced cytokine production [72]	*Cotinus coggygria* [70] *Butea frondosa* [73] *Gleditsia triacanthos* [73] *Quebracho Colorado* [73] *Curcuma longa* [73] *Rhus verniciflua* [73] *Acacia greggii* [73] *Acacia berlandieri* [73]

Morin	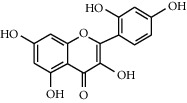	(1) Antioxidant properties [74] (2) Enhances pancreatic secretion of insulin [75] (3) Improves insulin resistance [76]	*Psidium guajava* [77] *Prunus dulcis* [78] *Chlorophora tinctoria* [78] *Maclura pomifera* [76]

## Data Availability

The data used to support the findings of this study are included within the article.
